# Extraction of a 20-year-old implanted permanent transfemoral dual-chamber pacemaker system

**DOI:** 10.1016/j.hrcr.2022.02.007

**Published:** 2022-02-24

**Authors:** Min Choon Tan, Arturo M. Valverde, Justin Z. Lee

**Affiliations:** Mayo Clinic, Phoenix, Arizona

**Keywords:** Electrophysiology, Lead extraction, Mechanical sheath, Transfemoral pacemaker, Dual-chamber pacemaker system

**Key Teaching Points**

• Transvenous extraction of a chronically implanted transfemoral pacemaker system using the mechanical sheath is a safe and feasible procedure with meticulous preprocedure preparation and appropriate extraction tools and technique.
• Risk stratification of the patient before the procedure, cardiothoracic and vascular surgery backup, blood preparation, superior vena cava (SVC) balloon, and intraprocedural transesophageal echocardiogram are crucial to ensure the safety of the procedure.
• Given that the SVC and inferior vena cava (IVC) are similar in size, the endovascular occlusion balloon should be able to be used in an inferior rescue approach. However, there are a few considerations for the differences between the potential impact of inflation of a balloon in the SVC compared to the IVC.

## Introduction

Lead extraction is increasingly performed to remove the implants owing to device infection or pacemaker lead malfunction. Permanent transfemoral pacemakers are rare owing to the risk of infection, atrial lead dislodgement, and chronic venous thrombosis.^1^ Reports of successful lead extraction of transfemoral dual-chamber pacemaker systems are limited.^2^ We describe a case of successful mechanical lead extraction of a 20-year-old transfemoral dual-chamber pacemaker system and discuss the procedural approach.

## Case report

**Figure 1:**
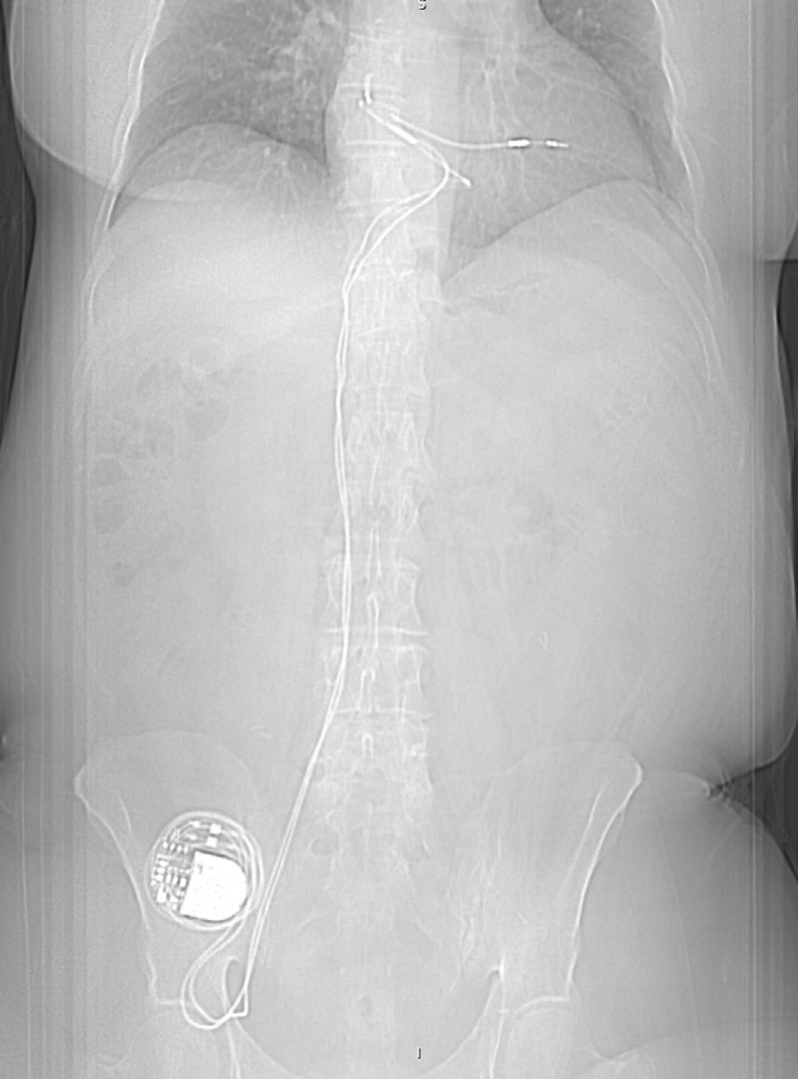
Radiographic image of permanent transfemoral dual-chamber pacemaker.

The patient is a 65-year-old woman with paroxysmal atrial fibrillation, right-side breast cancer in remission, complete heart block diagnosed 20 years ago status post permanent transfemoral dual-chamber pacemaker, who presented with atrial lead malfunction. She developed right-side breast cancer 20 years ago and underwent right-side mastectomy and chemotherapy. However, she also developed a complete heart block around that time. As she had a left-side Hickman catheter, a dual-chamber pacemaker was implanted via the right femoral vein approach (Figure 1). The Hickman catheter was subsequently removed, but the femoral pacemaker system was left in place. She had a generator change 10 years later. The patient had remission from her breast cancer but was later found to have symptomatic paroxysmal atrial fibrillation. This was managed with sotalol 120 mg administered orally twice a day.

Twenty years after the dual-chamber pacemaker was initially implanted, she was found to have atrial lead malfunction with intermittent undersensing of atrial activity. This was found after a 48-hour Holter monitor showed 19% of the recording as atrial fibrillation, with episodes of rapid rate. The Holter data were discordant with her device check, which showed only 0.1% atrial fibrillation burden even with atrial lead sensitivity programmed at the most sensitive setting. Pacemaker interrogation had a sensed sinus P wave at 0.7 mV. Her complete heart block was also found to have been only when she was undergoing breast cancer therapy. Since then, she has had recovery of her atrioventricular conduction and was no longer pacemaker dependent. However, she does have sinus node dysfunction and still requires atrial pacing.

The therapeutic options were then discussed with the patient, including continued clinical observation, abandoning the femoral pacemaker system and implanting a new system, or extraction of the transfemoral pacemaker system. Following shared decision-making, the decision was made for extraction of the transfemoral pacemaker system with reimplantation of a dual-chamber pacemaker system via the superior approach. An upper-extremity venogram was performed to confirm patency of the bilateral subclavian vein.

For the extraction procedure, the patient was placed under general anesthesia. Cardiothoracic surgery backup was available. Two units of packed red blood cells were prepared in the room. The endovascular occlusion balloon was also available if needed to be inflated for tamponade of any inferior vena cava (IVC) tear. Multiple vascular accesses were obtained, including in the right internal jugular vein, left femoral vein, and left femoral artery.

The pacemaker generator was at the right lower abdomen. The prior scar was reincised, and dissection was carried down to the pulse generator that was freed from the pocket. A second incision was required at the right inguinal area in order to free up the lead, as it was tunneled from the right inguinal area to the right lower abdomen. The lead was then disconnected from the generator and freed into the lower incision. A long 110 cm straight stylet was advanced into the right atrial lead and right ventricular lead, and the active fixation screw was successfully retracted from the right ventricular lead but not the right atrial lead.

**Figure 2:**
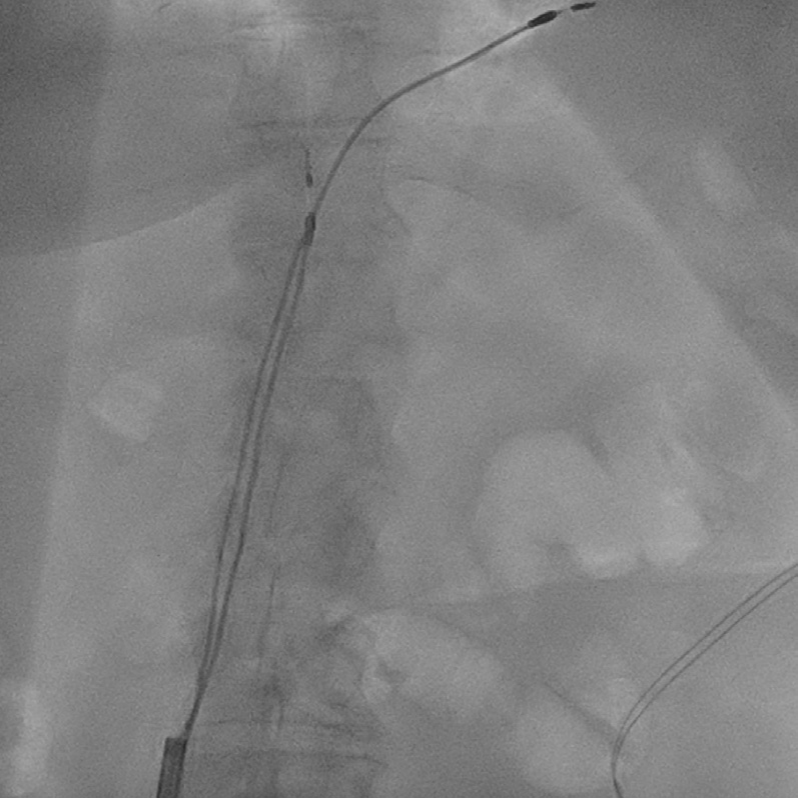
Extraction of transfemoral pacing leads with a mechanical sheath.

A locking stylet was then advanced into the lead lumen of the right atrial lead and the right ventricular lead. The 11F TightRail™ mechanical sheath (Spectranetics, Colorado Springs, CO) was used, starting with the right ventricular lead (Figure 2). There was evidence of multiple lead-lead binding sites in the IVC but predominantly over the hepatic region of the IVC. This required alternating the TightRail mechanical sheath between the right atrial and the right ventricular lead to free the leads from each other. With counter-traction at the level of the hepatic segment of the IVC, both leads were freed from the right atrium and right ventricle. They were then subsequently removed from the body. Transesophageal echocardiogram showed no pericardial effusion and no change in severity of tricuspid regurgitation. Subsequently, a new dual-chamber pacemaker system was implanted via the left subclavian approach.

## Discussion

In the patients with pacemaker implant via the subclavian approach, the use of lead extraction via femoral approach has been indicated in certain scenarios, such as after a failed primary approach via the implant vein or for removal of broken or cut leads with free ends.^3^ In contrast, reports of extraction of a transfemoral pacemaker system are limited. To our knowledge, this case will be the oldest femoral device extraction reported to date.

There has been data comparing the cap-and-abandon approach with extraction of sterile leads during device upgrade or replacement, with data showing extraction associated with lower risk of device infection.^4^ As recommended in the 2017 Heart Rhythm Expert Consensus, shared decision-making is important when considering whether to abandon or remove a lead.^5^ This case report showed that with meticulous preprocedure preparation and appropriate extraction tools and technique, similar to most extraction procedures, chronically implanted femoral leads could be extracted safely and successfully.

Preprocedural planning starts with risk stratification of the patient in order to determine whether the procedure should be performed in the operation room or device lab. Other important preparations include ensuring cardiothoracic and vascular surgery backup availability and that blood transfusion is ready in the room if needed. The superior vena cava (SVC) balloon should also be available to ensure that appropriate tamponade of any vena cava tear can be performed. An intraprocedural transesophageal echocardiogram is also helpful to detect any pericardial effusion during the intracardiac portion of the extraction procedure.

It is also important to ensure that the leads are freed and aligned with the direction of its course in the IVC for proper alignment of the extraction sheath. This will involve making an additional incision lower in the inguinal region and freeing the leads from its tunneled position in the lower abdomen. The mechanical sheath or laser sheath are the options for specialized extractions tools. In our case, we used the TightRail mechanical sheath. There is observational data showing lower risk of mortality with mechanical sheath compared to laser sheath.^6^ Furthermore, significant calcification and lead-to-lead binding was encountered, which will pose a challenge to the laser sheath. In our case, extensive lead-to-lead binding required alternating the mechanical sheath between right atrial and right ventricular leads to free the leads from each other. It is also worth noting that the working length of the 11F TightRail sheath is 47.5 cm, and this does permit its advancement from the femoral access point to the level of the cardiac border in our patient, who is 168 cm in height.

It is also important to consider either preinflation of the endovascular occlusion balloon or at least having a stiff rescue guidewire in place prior to the extraction.^7^ The endovascular occlusion balloon should be able to be used in an inferior rescue approach, given that the SVC and IVC are similar in size. There are a few considerations for the differences between the potential impact of inflation of a balloon in the SVC compared to the IVC. Firstly, there is a greater potential for hemodynamic instability with the occlusion, as the SVC only accounts for 35% of venous return while IVC is responsible for the remainder.^8^ Thus, during an emergency, it will then be difficult to ascertain hypotension from obstruction of venous return vs hypotension from bleeding. However, it can be theorized that the former would be a more acute hypotension compared to the latter. Doing a test inflation at the start of the procedure may help elucidate this. Secondly, if the balloon is inflated at the level of the renal veins, it may lead to increased hydrostatic pressure in the venous capillary system and acute kidney injury. Thirdly, prolonged inflation during an inferior rescue could lead to venous thrombosis. In our case, the decision was made to have the balloon ready but only advance the stiff wire and balloon if there is significant lead-to-IVC adherence and difficultly with the advancement of the mechanical sheath. In this case, it is reassuring that unlike the SVC (where there is an angulation that the sheath has to maneuver, which increases the risk of SVC tear), when the sheath is advanced from the femoral approach, the direction of the sheath is parallel to the course of the IVC. Nonetheless, for increased procedural safety, it is reasonable to consider at least having the stiff wire positioned.

## Conclusion

Transvenous extraction of a chronically implanted transfemoral pacemaker system using the mechanical sheath is a safe and feasible procedure. Thorough preprocedural and intraprocedural preparation is important to reduce the risk of procedural complications.
